# PD02 - Observational follow-up study with the Pan-European standard prick test to determine inhalant allergen sensitisation rates in a Greek population

**DOI:** 10.1186/2045-7022-4-S1-P2

**Published:** 2014-02-28

**Authors:** Anastasios P Konstantinopoulos, Dimitrios Karantoumanis, Maria Psomiadou, Eirini Roumpedaki, Anna Pananaki, Katerina Salavoura, Paraskevi Korovessi, Marianna Tziotou, Anastasia Karamouza, Nikolaus G Papadopoulos, Konstantina Piskou, Dimitrios Koutsalitis, Paraskevi Xepapadaki, Emmanouil Manousakis, G Nikolaos Papadopoulos

**Affiliations:** 1 Allergy Department, 2nd University Pediatric Clinic, University of Athens, Children’s Hospital “P. and A. Kyriakou”, Athens, Greece

## Introduction

Skin prick testing (SPT) is the standard method for diagnosing allergic sensitization. Correct diagnosis of inhalant allergies, requires knowledge of the most important inhalant allergen sensitizations. Few data are available regarding the prevalence of these sensitizations in Greece.

## Objective

The prospective assessment of the inhalant allergen sensitization rates in a Greek pediatric population.

## Methods

196 patients 3-18 years old (median age 10.3) who referred to our outpatient department with suspected reactions to inhalant allergens were included in the study (1.6/1 male to female ratio). Data were collected on the first 20 SPT performed every month for a total period of 12 months (April 2012 to March 2013) and are herein reported for children (<18), only. Evaluation for every skin prick test occurred after 15-20 min exposure, with positive results defined as a wheal ≥3 mm maximum diameter.

## Results

The seven allergens with the most common sensitization rate were revealed to be olea (52.3%), followed by Grass-mix (42.6%), cat (31.3%), mites [D. pteronissinus (31.8%), D. farinae (23.6%)], parietaria (24.6%), alternaria (19.5%). These were comparable with a previous evaluation of our population. Sensitization to ragweed and birch (14.9% each) allergens was also present, although these allergens do not exist in southern Greece.

13.3% of the subjects were not sensitized to any allergen tested. Single sensitization was revealed in 18.3%, the most prevalent being mites (6.1%), olive (4.6%) and grasses (4.1%). 48% were sensitized to four or more allergens.

## Conclusion

The application of the Pan-European panel may provide awareness for some inhalant allergen sensitizations that had not been examined in this population before. It also suggests that some sensitizations assessed through SPTs with natural extracts, may be due to cross-reactivity, highlighting the need for component resolved diagnosis.

